# The Relation Between Echocardiographic Epicardial Fat Thickness and CHA_2_DS_2_-Vasc Score in Patients with Sinus Rhythm

**DOI:** 10.21470/1678-9741-2020-0017

**Published:** 2020

**Authors:** Mesut Engin

**Affiliations:** 1 Department of Cardiovascular Surgery, University of Health and Sciences, Mehmet Akif Inan Training and Research Hospital, Sanliurfa, Turkey. E-mail: mesut_kvc_cor@hotmail.com

**Dear Editor,**

I have read the article by Aksoy et al.^[1]^, entitled “The Relation Between Echocardiographic Epicardial Fat Thickness and CHA2DS2-VASc Score in Patients with Sinus Rhythm”. First of all, I congratulate the authors for their invaluable contribution to the literature. On the other hand, I would like to clarify a point about the waist circumference (WC).

Obesity is one of the most important factors in cardiovascular diseases. Although body mass index (BMI) has been extensively investigated in cardiovascular diseases, WC and WC parameters have become more prominent in recent years^[2]^. In a large series of patients, it was shown that WC and its associated parameters were more correlated with myocardial infarction than BMI^[3]^. However, when the WC value is used alone, it is necessary to make a separate assessment for men and women^[4-6]^. In addition, this value is used as a risk factor for different values in different regions. For example, having a WC of >102 cm in men and of >88 cm in women is a risk factor for cardiovascular diseases in our country. A study by Girerd et al. included 2214 male patients who underwent coronary bypass surgery; the WC of >102 cm in these patients was found to be a risk factor for postoperative atrial fibrillation^[7]^.

In the study by Aksoy et al.^[1]^, the correlation between WC and epicardial adipose tissue was found to be mildly positive (r=0.184, *P*=0.02). However, WC was not defined as a gender-specific value by the authors^[1]^. Although this correlation is guiding us for future studies, I think that it may be more effective to evaluate patients according to their gender groups. Thus, the results of statistical analysis may change. Or this assessment can be performed using lipid accumulation product and/or visceral adiposity index and/or waist to height ratio, predictors of visceral fat^[8,9]^.
